# Induction of Ovarian Primordial Follicle Assembly by Connective Tissue Growth Factor CTGF

**DOI:** 10.1371/journal.pone.0012979

**Published:** 2010-09-24

**Authors:** Ryan Schindler, Eric Nilsson, Michael K. Skinner

**Affiliations:** Center for Reproductive Biology, School Biological Sciences, Washington State University, Pullman, Washington, United States of America; University of Dayton, United States of America

## Abstract

Primordial follicle assembly is a process that occurs when oocyte nests break down to form individual primordial follicles. The size of this initial pool of primordial follicles in part determines the reproductive lifespan of the female. Connective tissue growth factor (CTGF) was identified as a potential regulatory candidate for this process in a previous microarray analysis of follicle development. The current study examines the effects of CTGF and associated transforming growth factor beta 1 (TGFβ-1) on follicle assembly. Ovaries were removed from newborn rat pups and placed in an organ culture system. The ovaries treated with CTGF for two days were found to have an increased proportion of assembled follicles. CTGF was found to regulate the ovarian transcriptome during primordial follicle assembly and an integrative network of genes was identified. TGFβ-1 had no effect on primordial follicle assembly and in combination with CTGF decreased oocyte number in the ovary after two days of culture. Over ten days of treatment only the combined treatment of CTGF and TGFβ-1 was found to cause an increase in the proportion of assembled follicles. Interestingly, treatment with TGFβ-1 alone resulted in fewer total oocytes in the ovary and decreased the primordial follicle pool size after ten days of culture. Observations indicate that CTGF alone or in combination with TGFβ-1 stimulates primordial follicle assembly and TGFβ-1 can decrease the primordial follicle pool size. These observations suggest the possibility of manipulating primordial follicle pool size and influencing female reproductive lifespan.

## Introduction

Oocytes in newborn rodents are present in ‘nests’ that are composed of many adjacent oocytes with no intervening cells. Follicle assembly occurs in the first few days after birth in rodents. In humans, follicle assembly begins during mid-gestation near week 18 and continues into the third trimester [1], [2]. During the developmental process of follicle assembly primordial follicles are formed from oocyte nests [3], [4], [5], [6], [7]. The reproductive lifespan of a female is determined in part by the size of the primordial follicle pool generated [8]. Initially in the embryonic ovary oogonia undergo mitotic proliferation and then enter the first meiotic division to become oocytes. These unassembled oocytes are present directly adjacent to one another in nest structures which are surrounded by somatic cells (i.e. precursor granulosa cells) [3], [4], [5], [6], [7], [9]. The process of follicle assembly results in the breakdown of oocyte nests, due partially to apoptotic cell death of many of the oocytes. Somatic cells move into the nests and intersperse between the remaining oocytes [10], [11], [12]. An oocyte must be surrounded by an adequate number of pre-granulosa cells to form a primordial follicle [13], [14], [15]. The primordial follicles formed constitute a pool of follicles having oocytes arrested in prophase I of meiosis [3], [4], [5], [6]. Primordial follicles gradually leave the arrested pool by undergoing the primordial to primary follicle transition. After transition follicles as they grow either undergo apoptosis/atresia or the follicles ovulate. In humans, when the pool of follicles is depleted reproduction ceases and women enter menopause [16], [17], [18], [19], [20]. Some studies have suggested the possibility that new follicles with oocytes may form in adulthood [21], [22], [23], but the preponderance of literature suggests that a defined and finite pool of primordial follicles exists [24]. It is possible that if the size of the follicle pool could be manipulated the reproductive capacity and fertile lifespan of the organism may change.

Although several signal transduction and transcription factors have been shown to influence the primordial follicle pool [25], [26], [27], [28], [29], few extracellular signaling factors have been shown to have an effect on ovarian follicle assembly. Treatment of neonatal mice with activin resulted in an increase in the initial pool size [30]. It has been shown that both estrogen and progesterone slow the rate of follicle assembly [31]. This study also demonstrated that progesterone acts at least in part through an inhibition of oocyte apoptosis. Previous studies have demonstrated that apoptosis of oocytes is part of follicle assembly [9], [32]. The actions of progesterone were later found to be inhibited by tumour necrosis factor-alpha (TNFα) [33]. TNFα did not have an effect on the rate or percentage of assembled follicles, but promoted the apoptosis of oocytes. TNFα was found to block the inhibitory actions of progesterone and promote normal primordial follicle assembly [33]. Analysis of the inhibitory actions of progesterone on follicle assembly using a microarray analysis of the ovarian transcriptome demonstrated that progesterone promoted a dramatic up-regulation of connective tissue growth factor (CTGF), as well as an increase in the TGFβ family member TGFβ-3 [33].

The current study was designed to investigate the role of CTGF in primordial follicle assembly due to its altered gene expression during this process [33], [34]. Connective tissue growth factor (CTGF) has been shown to be expressed by multiple cell types and to have numerous functions. CTGF is well known for its roles in tissue remodeling [35] and extra-cellular-matrix formation [36]. Tissue remodeling and extracellular matrix are important for follicular development [37]. CTGF is the most studied member of the CCN family of proteins, which is composed of cysteine-rich protein 61 (CYR61, CCN1), CTGF (CCN2), nephroblastoma overexpressed gene (NOV, CCN3), and Wnt-inducible secreted proteins 1–3 (WISP-1, WISP-2, WISP-3), which are also known as CCN4 through CCN6 [38]. CTGF mRNA has been shown to be present in ovaries in pre-antral to preovulatory follicles in both rats and pigs [39], [40]. CTGF and transforming growth factor-beta (TGFβ-1) have been shown to modify the functions of each other. For instance, CTGF regulates connective tissue fibroblasts [41] and vascular smooth muscle cells [42] through a modulation of TGFβ-1 actions.

Transforming growth factor beta-one (TGFβ-1) has been shown to be present in both rat and human ovaries during follicle assembly [43], [44]. TGFβ-1, TGFβ-2, and TGFβ-3 are known to be expressed throughout adulthood in ovarian follicles. TGFβs have been shown to stimulate oocyte meiotic maturation in pubertal 23–25 day old rats [45]. In humans, TGFβ-1 has been localized primarily to oocytes during the period from 18–22 weeks of gestation [44]. In contrast, in 1–3 day old rats, TGFβ-1 has been localized primarily to granulosa cells [43]. The role of TGFβ-1 in neonatal ovary development has not been determined.

The present study is designed to determine if CTGF and/or TGFβ-1 affect the process of follicle assembly or alter primordial pool size. Since the expression of CTGF changes during follicle assembly, as shown in recent microarray studies [33], [34], the functional role of CTGF was investigated. Interestingly, CTGF was found to induce primordial follicle assembly while TGFβ reduced primordial follicle pool size.

## Materials and Methods

### Histology and Organ Cultures

Harlan Sprague-Dawley rats from Washington State University breeder colonies were used in this study. Female rat pups (<8 hours old) were euthanized and their ovaries were dissected. All procedures were approved by the Washington State University Animal Use and Care Committee (IACUC approval # 02568-014). Whole ovaries were cultured as previously described [46] on floating filters (.4 µm Millicell-CM, Millipore, Bedford, MD, USA) in .5 ml Dulbecco's modified Eagle's medium (DMEM)-Ham's F-12 medium (1∶1, vol/vol) containing 0.1% BSA (Sigma), 0.1% Albumax (Gibco BRL, Gaithersburg, MD, USA), 27.5 µg/ml transferrin, and 0.05 mg/ml L-ascorbic acid (Sigma) in a four-well culture plate (Nunc plate, Applied Scientific, South San Francisco, CA, USA) for two or ten days. The medium was supplemented with penicillin and streptomycin to prevent bacterial contamination. Ovaries were randomly assigned to treatment groups, with 1–3 ovaries per floating filter. Wells were treated every two days with recombinant connective tissue growth factor (CTGF) (Peprotech, Rocky Hill, NJ, USA) at 500 ng/ml, transforming growth factor beta 1 (TGFβ-1) (R&D Systems, Minneapolis, MN, USA) at 50 ng/ml, a combination of both CTGF and TGFβ-1, or progesterone (P_4_) (Sigma) at 10^−6^ M. After culture, ovaries were fixed in Bouin's fixative (Sigma) for two hours. Ovaries were then embedded in paraffin, sectioned at 3 µm and stained with hematoxylin/eosin for use in morphological analysis.

### Morphological Analysis

The number of oocytes at each developmental stage was counted and the number for each developmental stage was averaged across two consecutive sections that had the largest ovarian cross section. The oocyte nucleus had to be visible for an oocyte to be counted. Normally, between 100 and 300 oocytes were present in each cross-section. Oocytes were microscopically examined and were classified as being not yet assembled into follicles (i.e. the oocyte was part of an unassembled oocyte nest), as primordial (stage 0) or as one of the developing pre-antral stages (stages 1–4) as described previously [31], [47]. Unassembled oocytes are recognized from being grouped together without any intervening stromal cells. As assembly occurs, the surrounding pre-granulosa cells and the developing basement membrane around the follicle provide a more distinct and visible division of one oocyte from another. Primordial follicles consist of an oocyte partially or completely surrounded by a single layer of squamous pre-granulosa cells. Developing (stages 1–4) follicles contain successively more cuboidal granulosa cells in layers around the oocyte [4], [5], [46], [47]. Total oocyte numbers per ovarian cross-section were divided by the area (mm^2^) of the cross-section to account for any differences in ovary size between individuals.

### Apoptosis Assay

The degree of oocyte atresia (*i.e.* apoptosis) was measured with the *in situ* cell death detection kit (Roche Applied Science, Indianapolis, IN). Briefly, neonatal ovaries were cultured for 2 days, at which time follicular assembly is readily observed. Then cultured ovaries were fixed, embedded in paraffin, and sectioned. Paraffin embedded slides were then treated as per instructions in the apoptosis detection kit. Use of terminal deoxynucleotidyl transferase-mediated deoxyuridine triphosphate nick end-labeling (TUNEL) *in situ* caused polymerization of fluorescein-labeled nucleotides to the free 3′ ends of DNA that had been cleaved during apoptosis. Therefore, fluorescein-stained cellular nuclei had multiple breaks in their DNA and were undergoing apoptosis at the time of fixation.

### Immunohistochemistry

Ovary sections from cultured postnatal day 0 (P0) ovaries (cultured for 2 days) were immunostained as described previously [48], for the presence of CTGF using anti-CTGF primary antibody (anti-CTGF rabbit IgG, 1 µg/ml; Santa Cruz Biotechnology, Santa Cruz, CA, USA). Briefly, 3 µm sections were deparaffinized, rehydrated through a graded ethanol series, boiled in 10 mM sodium citrate buffer, quenched in 3% hydrogen peroxide/20% methanol and 0.1% Triton-X solution, and then blocked with 10% goat serum (normal goat serum; Vector Laboratories Inc., Burlingame, CA, USA) for 20 min prior to incubation with 1 µg/ml CTGF antibody (R&D Systems) for 12 h at 4°C. The sections were then washed with PBS and incubated with 1∶300 diluted biotinylated secondary antibody for 45 min (goat anti-rabbit IgG; Vector Laboratories Inc.), washed again, and incubated with streptavidin peroxidase (Zymed, San Francisco, CA, USA) prior to color development with a DAB peroxidase substrate kit (Vector Laboratories Inc). Following development, the sections were dehydrated, coverslips mounted with xylene-based medium (Cytoseal-XYL; Richard Allan Scientific, Kalamazoo, MI, USA), and analyzed at 200×, 400×, and 1000× magnification using light microscope. Negative control experiments were performed using a non-specific primary antibody at 1 µg/ml (rabbit IgG; Sigma) and a negative control of an irrelevant anti-SRY antibody at 1 µg/ml (anti-SRY rabbit IgG, 1 µg/ml; Santa Cruz Biotechnology, Santa Cruz, USA).

### RNA isolation and purification

Samples of 2–3 pooled control on treated ovaries were stored in TRIZOL (Invitrogen) at –80°C until RNA extraction following the manufacturer's protocol. High quality RNA samples were assessed with gel electrophoresis and required a minimum OD_260/280_ ratio of 1.8. Three samples each of control and treated ovaries were applied to microarrays.

### Microarray Analysis

mRNA processing and hybridization were performed at Genomics Core Laboratory, Center for Reproductive Biology, Washington State University, Pullman, WA using standard Affymetrix reagents and protocol. Briefly, mRNA was transcribed into cDNA with random primers, from the later, cRNA was transcribed, and from that, single-stranded sense DNA was synthesized which was fragmented and labeled with biotin. Biotin-labeled fragmented ssDNA was then hybridized to the Rat Gene 1.0 ST microarrays containing more than 27,000 transcripts (Affymetrix, Santa Clara, CA, USA). Hybridized chips were scanned on Affymetrix Scanner 3000. CEL files containing raw data were then pre-processed and analyzed with Partek Genomic Suite 6.5 beta software (Partek Incorporated, St. Louis, MO) using RMA, GC-content adjusted algorithm. The signals from 11 probe sets for each transcript were combined to give a single value. Lists of differentially expressed genes treatment were generated using following cut off criteria: signal ratio Control/Treatment >1.20 or <0.83, mean difference for un-logged signals between control and treatment >10, t-test p-values <0.05. p-values were generated in 2-way ANOVA, blocking for organ culture date batch effects.

CEL files (MIAME compliant raw data) from this study have been deposited with the NCBI gene expression and hybridization array data repository (GEO, http://www.ncbi.nlm.nih.gov/geo, #GSE22096) and can be also accessed through www.skinner.wsu.edu. For genes annotation, Affymetrix annotation file RaGene1_0stv1.na30.rn4.transcript.csv was used unless otherwise specified. Generation of affected KEGG pathways (Kyoto Encyclopedia for Genes and Genome, Kyoto University, Japan) used Pathway-Express, a web-based tool freely available as part of the Onto-Tools (http://vortex.cs.wayne.edu) [49].

Previous studies have demonstrated that microarray data are validated with quantitative PCR data [34], [50]. Due to the presence of 11 different oligonucleotide sets for each specific gene being used on the microarray versus only a single primer set for a gene in a quantitative PCR, the microarray is more effective at eliminating false positive or negative data and provides a more robust quantification of changes in gene expression.

### Statistical Analysis

Treatment groups are compared using analysis of variance (ANOVA) followed by comparative t-tests where appropriate. Groups were considered statistically significant with P≤0.05. All statistics were calculated using GraphPad Prism version 5.0b for Macintosh, GraphPad Software, San Diego, CA, USA.

## Results

Data from a previous microarray and ovarian transcriptome analysis demonstrated the relative expression of TGFβ-1, 2, and 3 were highest in unassembled oocytes [34]. The inhibitory actions of progesterone on primordial follicle assembly was found to coincide with a dramatic increase in CTGF gene expression [33]. CTGF and TGFβ-3 are secreted growth factors that showed a stimulation by progesterone during follicle assembly. TGFβ-3 and TGFβ-1 bind to the same receptor, but only TGFβ-1 was commercially available. Therefore, CTGF and TGFβ-1 were selected as candidates to investigate experimentally as being involved in the regulation of primordial follicle assembly.

**Figure 1:**
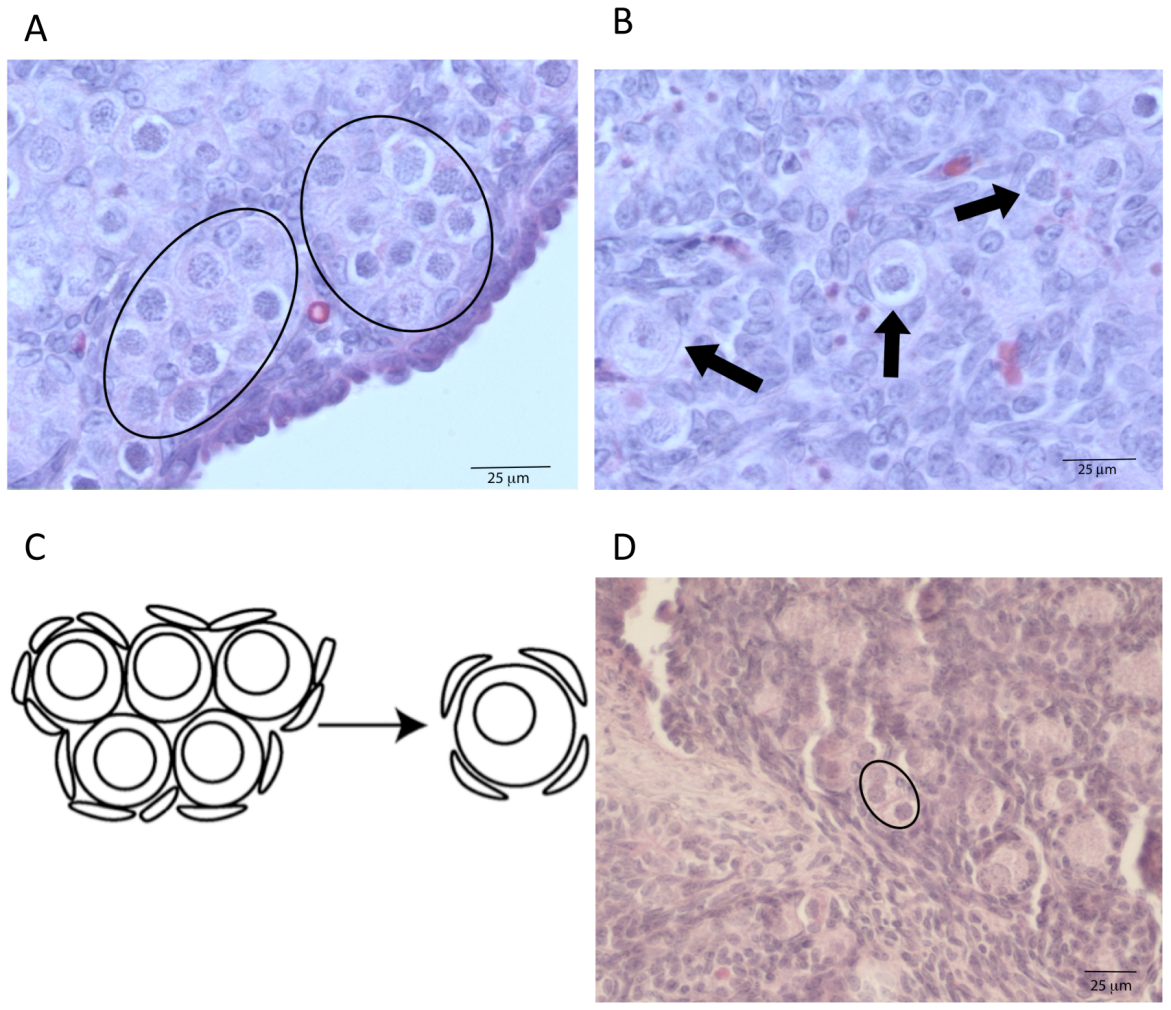
Follicle assembly in rat ovaries begins just after birth. Oocytes are initially present only in nests, with few or no intervening cells, (A) and the left half of (C). The circle outlines in (A) indicate the locations of two nests. Follicle assembly results in many of these oocytes undergoing apoptosis with the surviving oocytes developing into primordial follicles, indicated with arrows, (B) and the right half of (C). (A) P-0 ovary. (B) P-0 ovary after culture for 2 days. (D) shows a zero day ovary that has been cultured for ten days and has much smaller nests that contain fewer oocytes, indicated in circle.

**Figure 2:**
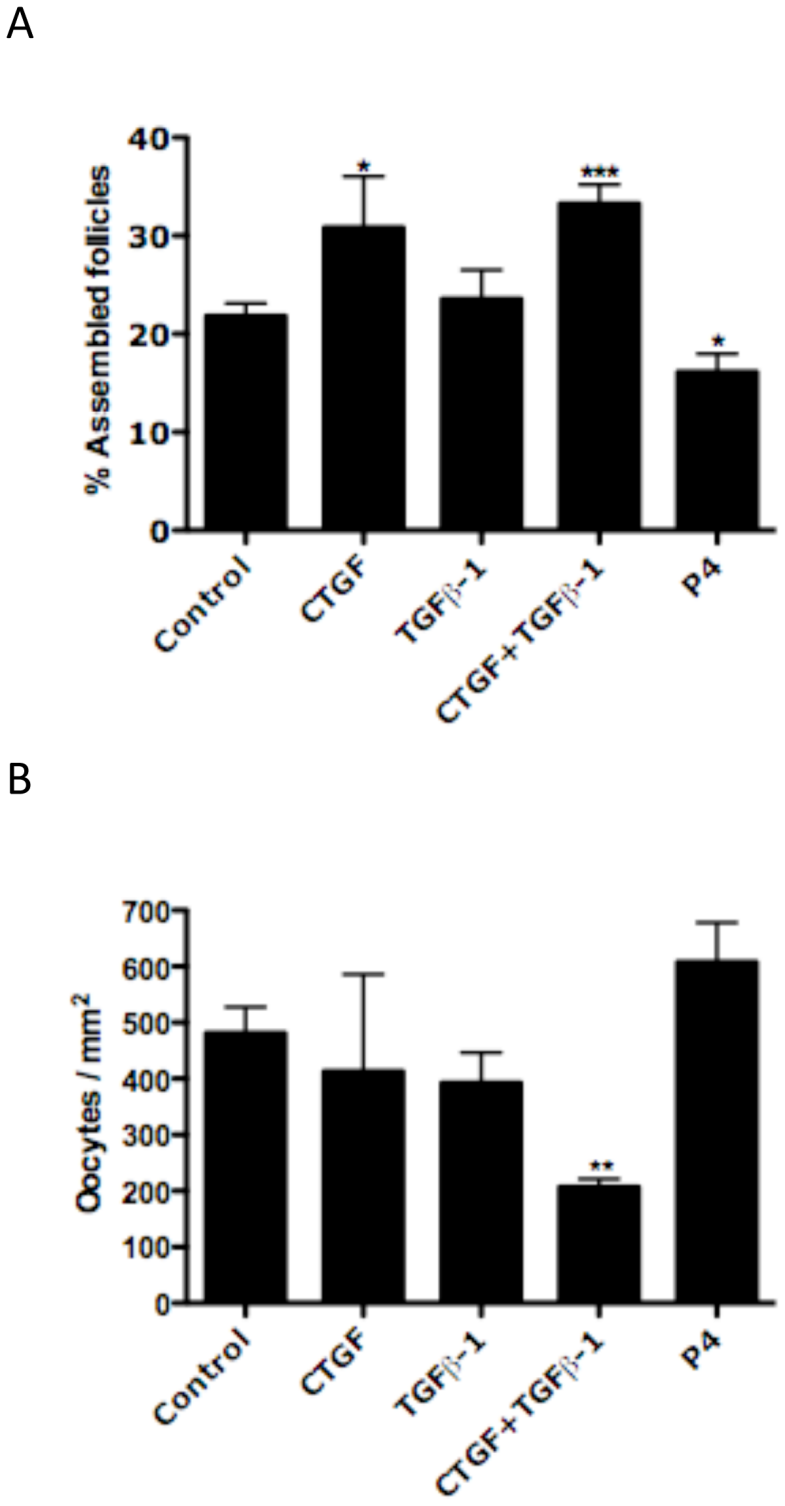
Effect of CTGF and TGFβ-1 treatment on follicle assembly in 0-day-old ovaries cultured for two days. (A) Effect of CTGF, TGFβ-1 and progesterone (P_4_) treatment on follicle assembly. Data are presented as the percentage of assembled oocytes (mean ± S.E.M.) pooled from eleven separate experiments (n = 4–16 per treatment group). One-way ANOVA showed a significant (P<0.005) effect of treatment. Bars with asterisks indicate the P-value in comparative pooled t-test vs. the control group. (*)P≤0.05 and (***)P≤0.001. (B) Effect of CTGF and TGFβ-1 treatment on oocyte density in ovaries cultured for two days. The total number of oocytes in each section was divided by the cross-sectional area of that ovary. Data are presented as the oocytes per square millimeter (mm^2^) (mean ± S.E.M.) pooled from eleven separate experiments (n = 4–16 per treatment group). One-way ANOVA showed a significant (P<0.05) effect of treatment. Bars with asterisks indicate the P-value in comparative pooled t-test vs. the control group. (**)P≤0.01.

The 0-day old rat ovary is composed almost entirely of nests of unassembled oocytes (Figure 1A). In the rodent by 4 days of age the follicle assembly process is in large part completed, and the oocytes have assembled into primordial follicles (Figure 1B, 1C). Therefore, the ovaries can be caught in an intermediate stage of assembly by using 0 day ovaries and culturing for 2 days. Ovaries from newborn 0-day-old rat pups were placed onto a floating filter organ culture system and treated with either 50 ng/ml transforming growth factor beta one (TGFβ-1), or 500ng/ml connective tissue growth factor (CTGF), or a combination of TGFβ-1 and CTGF, or 10^−6^M progesterone (P_4_), or were left untreated as controls. After two days of culture, the ovaries were fixed, sectioned and stained for morphological analysis. The relative proportion of assembled follicles was compared across treatment groups (Figure 2A). Treatment with CTGF was found to significantly increase the percentage of assembled follicles. As seen in previous studies [31], progesterone decreased the proportion of assembled follicles over 2 days. Response to progesterone was used as a positive control for these experiments. TGFβ-1 had no effect alone. The combined treatment of CTGF and TGFβ-1 was not different from CTGF alone. Observations indicate the presence of TGFβ-1 did not have a direct effect on the percentage of assembled follicles (Figure 2A).

For each ovary section counted the cross sectional area was determined using ImageJ [51]. The total number of oocytes in each ovary was normalized by its area (mm^2^) and the oocyte density for each ovary compared between treatment groups (Figure 2B). There was no difference in ovarian cross-sectional area between treatment groups (data not shown). Interestingly, the combined CTGF and TGFβ-1 treatment significantly decreased the oocyte density compared to controls. No other treatment groups had any significant effect. Therefore, the combination of CTGF and TGFβ-1 appeared to decrease the initial oocyte cohort/pool in the ovary after two days of culture.

**Figure 3:**
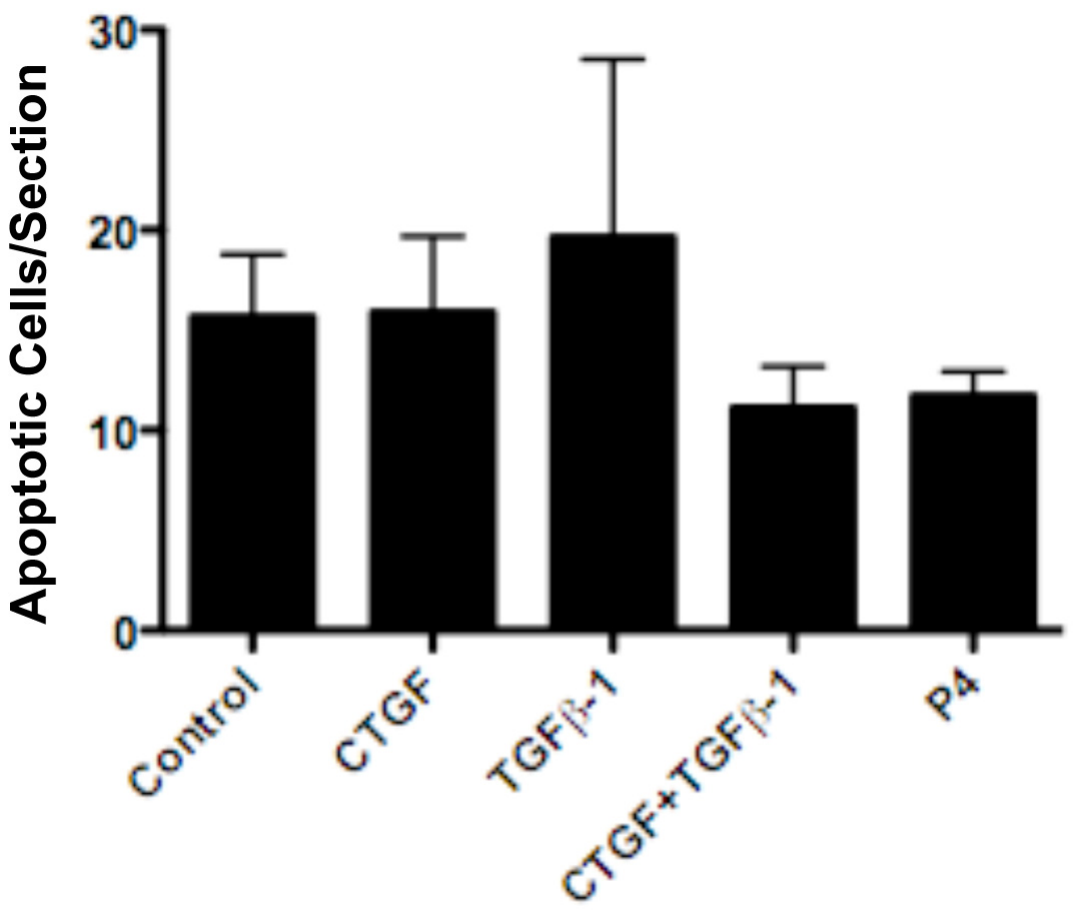
Effect of CTGF, TGFβ-1 and progesterone (P_4_) treatments on oocyte apoptosis in ovaries cultured for two days. After culture ovaries were fixed, sectioned and an apoptosis assay (TUNEL) performed on the sections. Data are presented as the number of apoptotic cells per cross section (mean ± S.E.M.) pooled from four separate experiments (n = 4–12 per treatment group). One-way ANOVA showed no significant (P = .6746) effect of treatment.

Oocyte apoptosis was investigated since the primordial follicle assembly process involves programmed cell death of the oocytes. The level of apoptosis was measured with TUNEL assays of DNA fragmentation. The number of apoptotic oocytes per section for each treatment group were compared (Figure 3). No significant alteration in oocyte apoptosis was observed in any treatment group. Therefore, the actions of CTGF did not appear to involve altered oocyte apoptosis at 2 days of culture.

**Figure 4:**
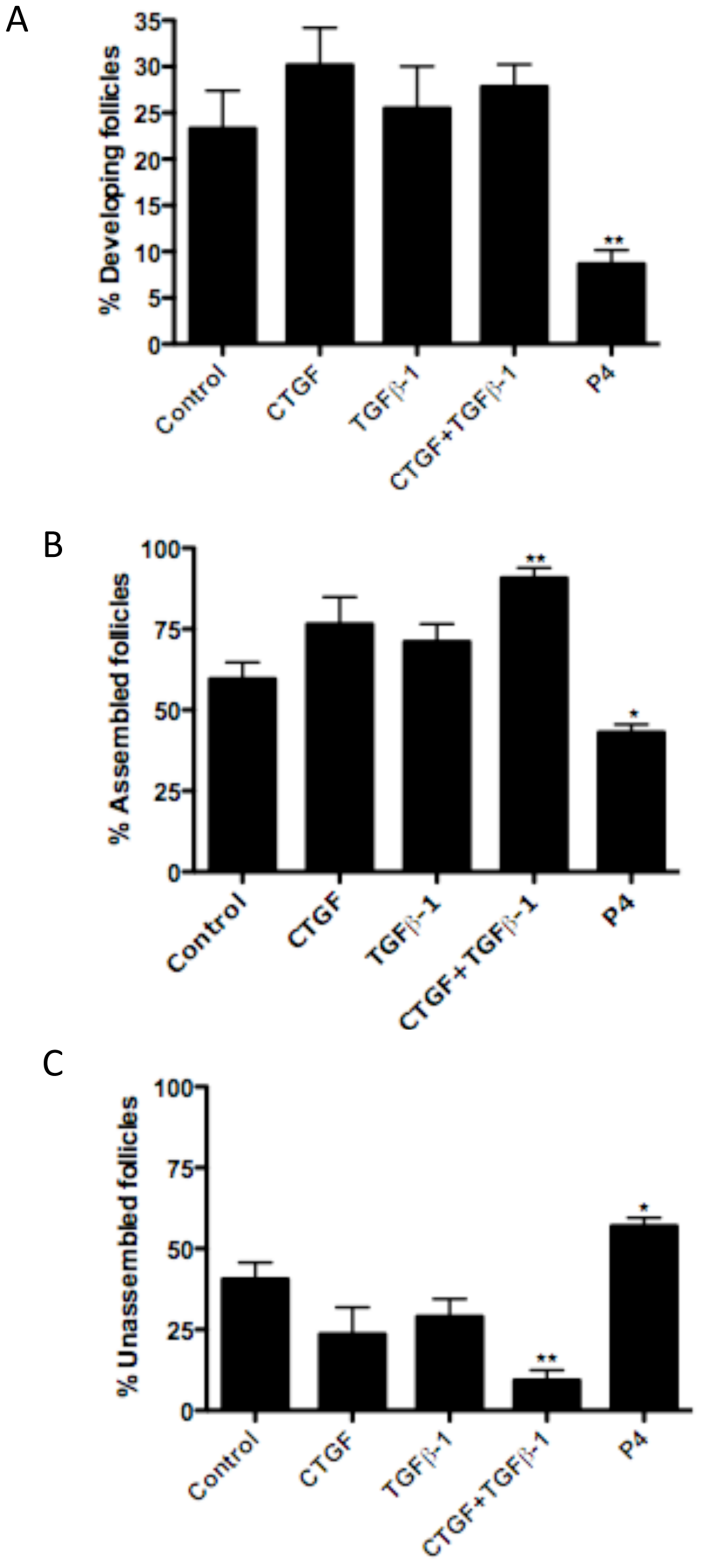
Effect of CTGF and TGFβ-1 treatments on follicle assembly in ovaries cultured for ten days. Data are presented as the percentage of (A) developing follicles (i.e. those that have undergone follicle transition), (B) assembled follicles (which include developing follicles), and (C) unassembled follicles (mean ± S.E.M.) pooled from six separate experiments (n = 6–13 per treatment group). One-way ANOVA showed a significant (P<0.05) effect of treatment. Bars with asterisks indicate the P-value in comparative pooled t-test vs. the control group. (*)P<0.05, (**)P<0.01.

**Figure 5:**
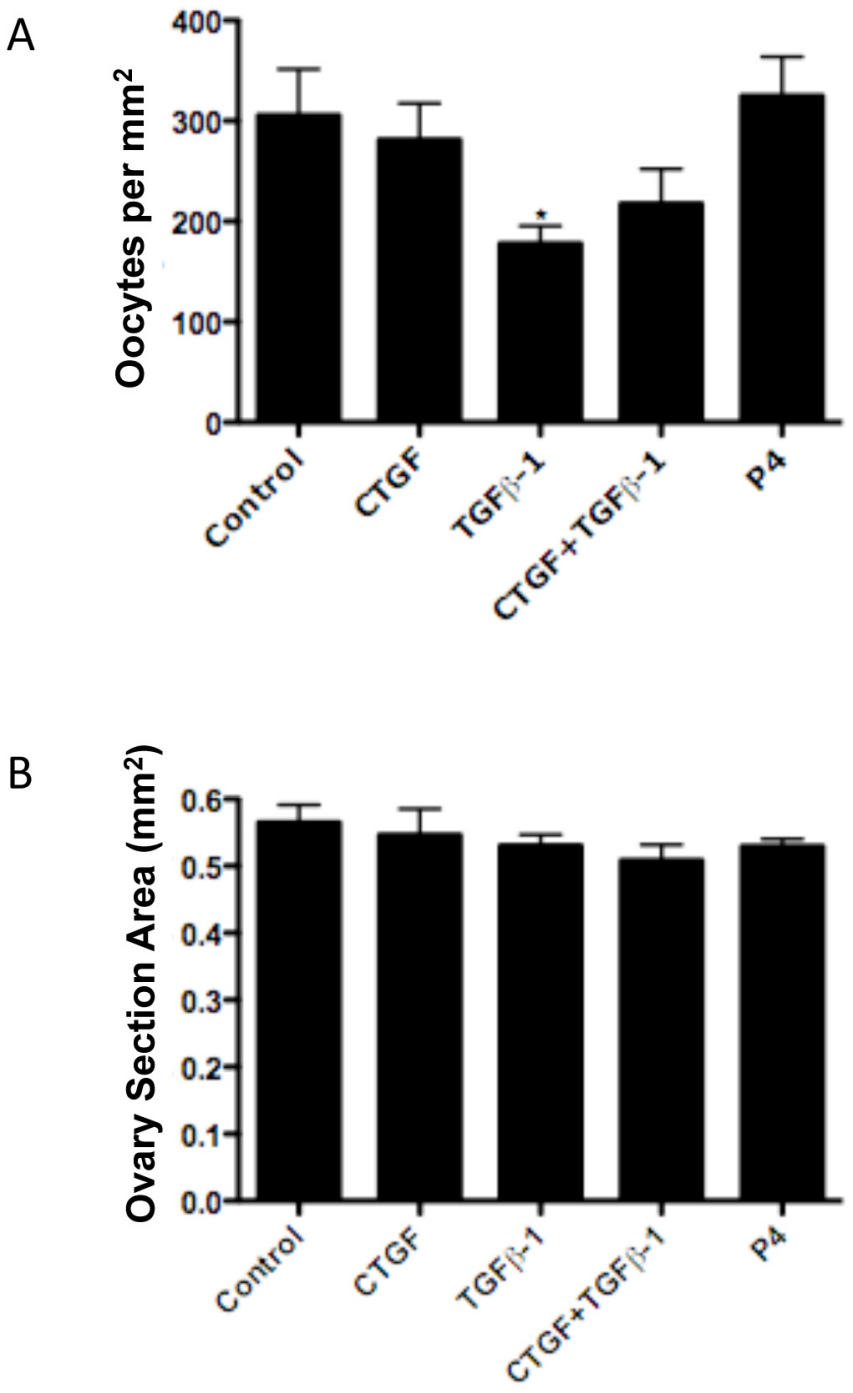
Effect of CTGF, TGFβ-1 and progesterone (P_4_) treatment on follicle assembly in ovaries cultured for ten days. Data area presented as (A) the total number of oocytes per cross sectional area (mm^2^) or (B) total cross sectional area (mm^2^) (mean ± S.E.M.) pooled from six separate experiments (n = 6–13 per treatment group). One-way ANOVA showed a significant (P<0.05) effect of treatments in (A). Bars with asterisks indicate the P-value in comparative pooled t-test vs. the control group.

The effects of the growth factors on the primordial follicle pool size used a 10-day ovary culture system. The combined treatment of CTGF and TGFβ-1 was found to increase the proportion of assembled primordial follicles and decrease the proportion of unassembled follicles (Figure 4B and 4C). Progesterone (P_4_) was found to decrease the percentage of assembled and developed follicles (Figure 4A and 4B) and increase the percentage of unassembled follicles compared to controls (Figure 4C). The unassembled follicles present were small clusters of 2–3 oocytes as shown in Figure 1D. The oocyte density and ovarian cross-sectional area in each ovary were compared across treatments (Figure 5). The TGFβ-1 treatment group showed a significant decrease in the number of oocytes (Figure 5A). In contrast, the total ovary section area (i.e. size) was not affected by any treatment (Figure 5B). The effects of P_4_ and CTGF on the 10-day cultures appeared to be due to altered unassembled follicle populations and not a change in pool size. However, the effects of TGFβ1 alone appears to be due to a reduced follicle pool size.

**Figure 6:**
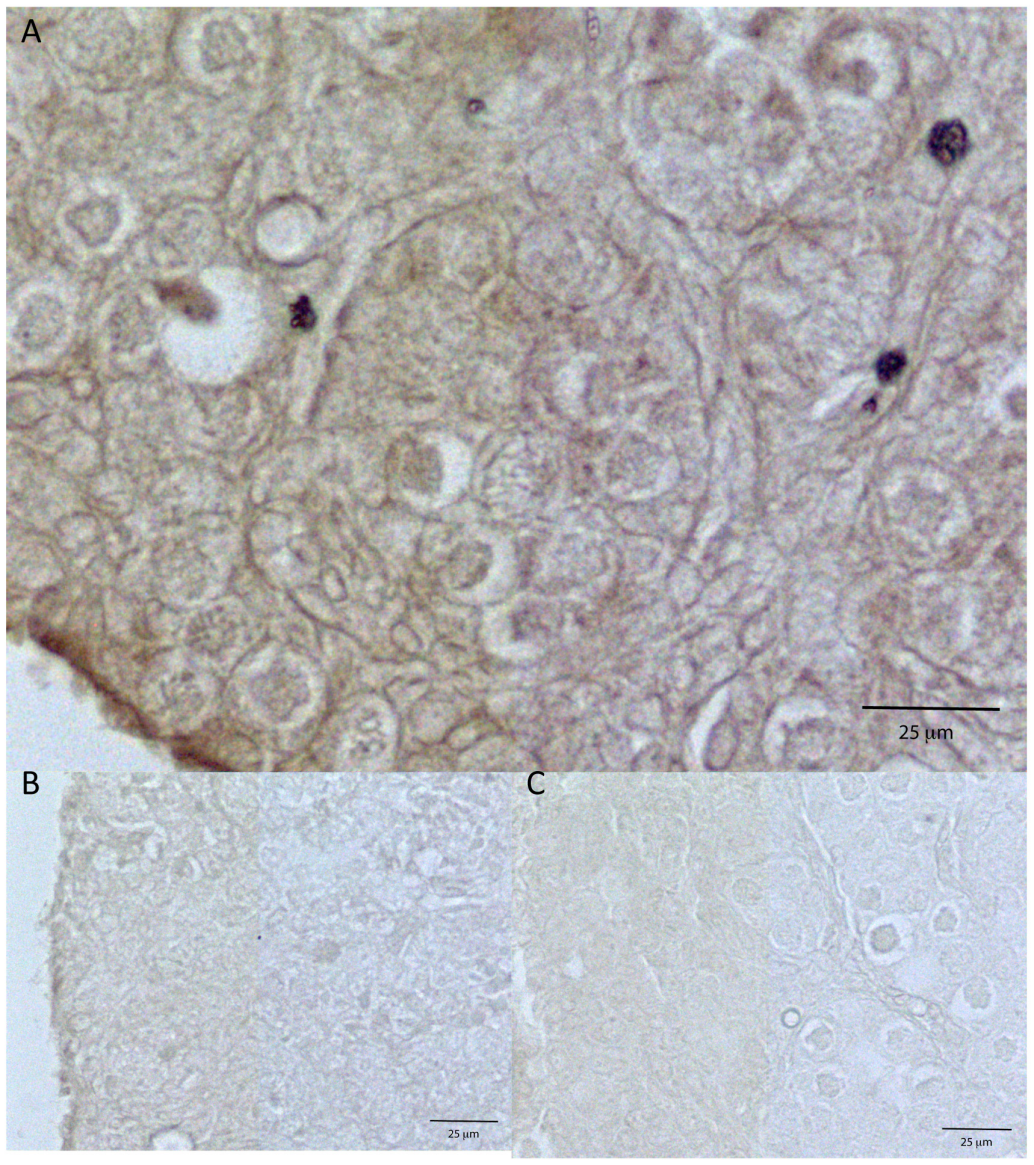
Localization of CTGF in cultured ovaries. Ovaries were dissected from zero day old rat pups and cultured for two days. Immunohistochemistry was performed on ovary sections. Only untreated ovaries were selected for this procedure. The primary antibodies used were anti-CTGF (A), negative control non-specific goat IgG (B), and negative control anti-SRY IgG (C).

Immunohistochemistry was performed on untreated P0 ovaries that had been cultured for two days to determine where CTGF was localized (Figure 6). CTGF was localized to the basement membranes of both nests and more developed follicles. As ovaries in culture undergo growth and development it is expected that the cell type distribution of ligand and receptor will be similar to that *in vivo* [39], [40]. Therefore CTGF appears to be localized to basement membranes and expressed throughout the ovary. Neither use of non-specific IgG as a primary antibody nor an irrelevant anti-SRY antibody resulted in any specific staining in the ovary.

**Figure 7:**
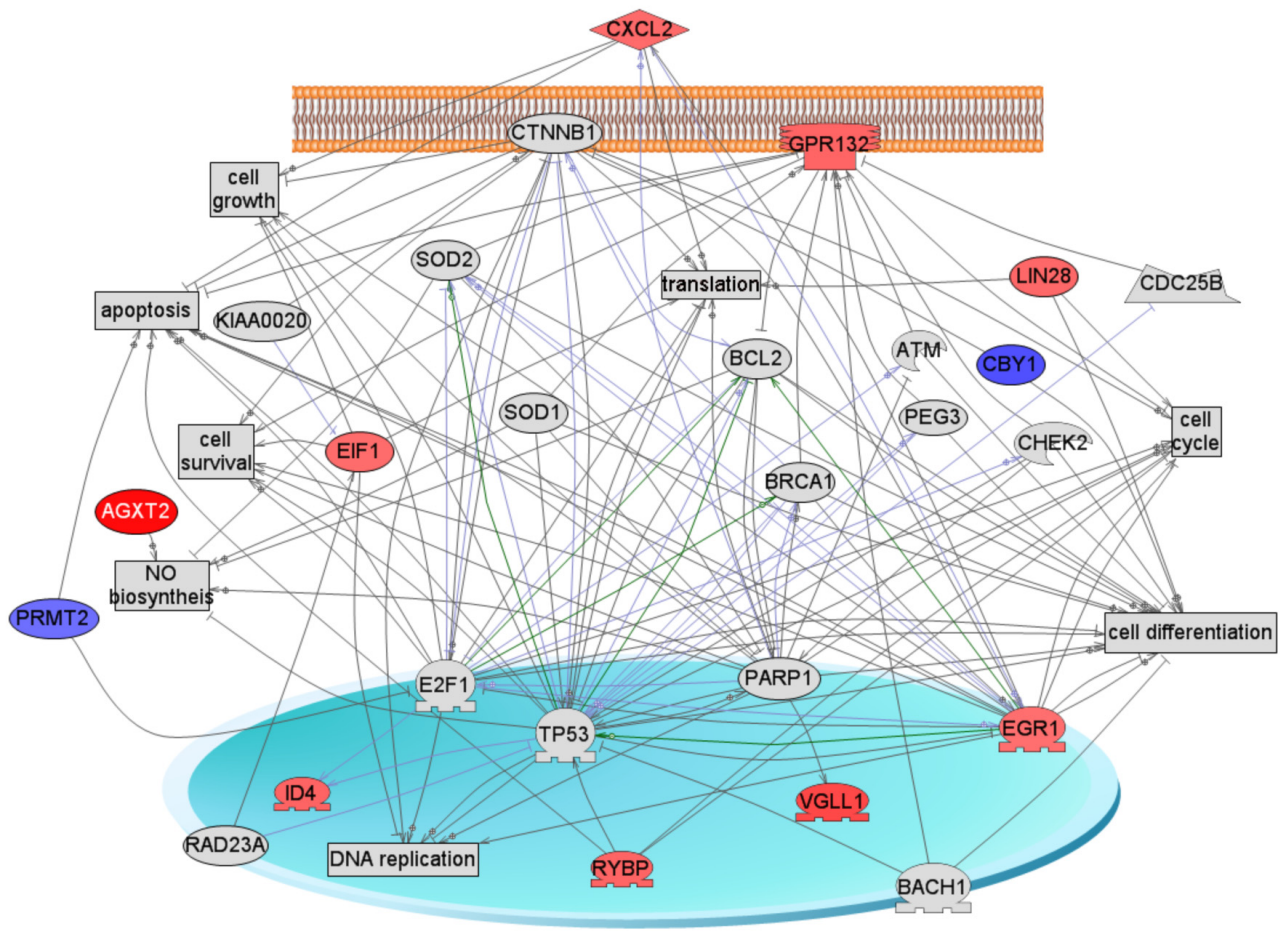
Scheme of shortest connections to cellular processes for the 76 differentially expressed genes obtained by global literature analysis (Pathway Studio, Ariadne). Only 11 connected genes from the list of 76 are shown – colored red or blue; the rest are not connected and not shown. Red color represents up-regulated genes, blue color – down-regulated genes, grey color means that gene/protein is not from the list of 76. Arrows with plus sign show positive regulation/activation, arrows with minus sign – negative regulation/inhibition; grey arrows represent regulation, lilac - expression, and green - promoter binding. Node shapes code: oval and circle – protein; crescent – protein kinase and kinase; diamond – ligand; irregular polygon – phosphatase; circle/oval on tripod platform – transcription factor; ice cream cone – receptor, rectangle - cell processes.

The molecular actions of CTGF on primordial follicle assembly were investigated with a microarray analysis of the ovarian transcriptome. Ovaries from newborn 0-day-old rat pups were cultured for 1 day of treatment prior to collection for analysis. Morphological analysis of these ovaries demonstrated negligible effects on follicle morphology and assembly after only 1 day of treatment (data not shown). This allows an analysis of CTGF actions on the ovarian transcriptome independent of morphological changes to the ovary. CTGF was found to alter the expression of 76 genes with 33 having an increase in expression and 43 having a decrease in expression. A list of these known genes in their appropriate functional categories is presented in Table 1. The presence of Cxcl2 suggests this chemokine may also be involved in follicle assembly. Interestingly, several olfactory receptors were found to be regulated in the ovary by CTGF treatment, Table 1 and Supplemental Table S1. In addition, an integrated gene network analysis demonstrated 11 regulated genes from this list formed a gene network with appropriate interactions with critical cellular processes (Figure 7). The most abundant functional classes of regulated genes were cell differentiation, cell cycle and apoptosis. Therefore, insights into the molecular actions of how CTGF promotes follicle assembly are provided.

**Table 1:** CTGF Regulated Genes During Primordial Follicle Assembly.

*Gene Symbol*	*GenBank_Reference Sequence*	*mean_diff (CTGF-Con)*	*Ratio CTGF/Con*	*Gene Title*
**Apoptosis**				
Rybp	NM_001107879	74	1.22	RING1 and YY1 binding protein
**Cell Cycle**				
Egr1	NM_012551	26	1.20	early growth response 1
**Cytoskeleton & ECM**				
Mybpc1	X90475	−14	0.82	myosin binding protein C, slow type
**Development**				
Id4	NM_175582	12	1.23	inhibitor of DNA binding 4
Tsx	NM_019203	8	1.32	testis specific X-linked gene
Vgll1	XM_001058701	6	1.31	vestigial like 1 (Drosophila)
**Epigenetics**				
Prmt2	NM_001025144	−31	0.84	protein arginine methyltransferase 2
**Growth Factors**				
Cxcl1	NM_030845	35	1.21	chemokine (C-X-C motif) ligand 1 (melanoma growth stimulating activity, alpha)
**Immune Response**				
LOC683127	XM_001064581	−32	0.77	similar to CD209a antigen
**Metabolism**				
Agxt2	NM_031835	14	1.54	alanine-glyoxylate aminotransferase 2
Ard1b	NM_001024742	12	1.20	ARD1 homolog B (S. cerevisiae)
Pdia5	NM_001014125	26	1.33	protein disulfide isomerase family A, member 5
**Proteolysis**				
Spink5l1	NM_001008874	4	1.22	Kazal type serine protease inhibitor 1
**Receptors & Binding Proteins**				
Ly49s6	NM_001009488	−7	0.79	Ly49 stimulatory receptor 6
Olr1226	NM_001000442	−10	0.84	olfactory receptor 1226
Olr46	NM_001001001	3	1.23	olfactory receptor 46
Olr630	NM_001001059	5	1.31	olfactory receptor 630
Olr684	NM_001001378	5	1.21	olfactory receptor 684
Olr690	NM_001000569	5	1.21	olfactory receptor 690
Olr727	NM_001000619	−5	0.76	olfactory receptor 727
Olr901	NM_001000057	−8	0.78	olfactory receptor 901
Olr920	NM_001001356	13	1.42	olfactory receptor 920
RGD1559730	AAHX01105426	−7	0.78	similar to putative pheromone receptor (Go-VN4)
Vom2r12	NM_001099488	−3	0.83	vomeronasal 2 receptor, 12
**Signaling**				
Cby1	NM_145676	−23	0.78	chibby homolog 1 (Drosophila)
Gpr132	NM_001170595	6	1.22	G protein-coupled receptor 132
MGC109340	BC092634	−25	0.83	similar to Microsomal signal peptidase 23 kDa subunit (SPase 22 kDa subunit) (SPC22/23)
Minpp1	NM_019263	20	1.28	multiple inositol polyphosphate histidine phosphatase 1
LOC690930	XM_001078213	−5	0.84	similar to membrane-spanning 4-domains, subfamily A, member 6B
**Transcription**				
Dnaja4	NM_001025411	7	1.24	DnaJ (Hsp40) homolog, subfamily A, member 4
Dnajc30	NM_001109024	−16	0.72	DnaJ (Hsp40) homolog, subfamily C, member 30
Hint1	BC168732	−7	0.77	histidine triad nucleotide binding protein 1
RGD1564534	BC111405	16	1.50	similar to CHCHD4 protein
**Translation & Protein Modification**				
Eif1	NM_001105837	13	1.20	eukaryotic translation initiation factor 1
Lin28	NM_001109269	31	1.21	lin-28 homolog (C. elegans)
LOC298509	XR_007384	−6	0.82	similar to 60S ribosomal protein L21
LOC688243	ENSRNOT00000045033	−6	0.82	similar to Nucleolin (Protein C23)

## Discussion

Previous ovarian transcriptome analysis demonstrated that TGFβ-1 is highly expressed during the follicle assembly process when compared to ovaries containing primordial or primary follicles [34]. In addition, the ability of progesterone to inhibit follicle assembly resulted in a dramatic increase in expression of CTGF [33]. CTGF and TGFβ-3 were among the few growth factors shown to be regulated. TGFβ-1 and TGFβ-3 act on the same receptor complex to stimulate the same signaling cascade. CTGF and TGFβ-1 are also known to regulate each others' functions [41], [42]. Therefore, CTGF and TGFβ-1 were investigated as potential regulators of primordial follicle assembly utilizing whole ovary organ cultures.

Observations from the two-day ovary culture experiments demonstrates that CTGF promotes primordial follicle assembly. TGFβ-1 treatment was not found to have an effect alone. The combined treatment of CTGF and TGFβ-1 stimulated follicular assembly to the same degree as CTGF alone. Therefore, TGFβ-1 had no effect on the proportion of oocytes assembled into follicles after two days of culture. Treatment with progesterone (P_4_) inhibited follicle assembly as previously described [31], and was used as a positive control in these experiments. Analysis of oocyte density demonstrated that the combined treatment of CTGF with TGFβ-1 induced a significant decrease in oocyte number. This suggests that the two factors synergize to modify oocyte number and demonstrates that initially it is possible to manipulate follicle pool size. One possible way to stimulate follicular or ovarian development and decrease pool size is by increasing apoptosis of the oocytes. This was tested with a cellular apoptosis assay, but no significant effect of any treatment was demonstrated. Therefore, CTGF and the combined CTGF & TGFβ-1 treatment groups appear not to act through altering apoptosis. However, it is possible that a difference in apoptosis rates may not be detectable at two days of culture. Further studies with shorter or longer culture periods are needed to clarify if there is a period at which CTGF and TGFβ-1 affect apoptosis. Alternatively, CTGF may modify the extra-cellular matrix (ECM) of either pre-granulosa cells or the oocytes. In summary, after two days of ovary culture CTGF was found to induce primordial follicle assembly while a combined CTGF and TGFβ-1 treatment initially reduced the pool of oocytes in the ovary.

Morphologically, the basement membranes of the nests are degraded and new membranes around primordial follicles are formed during follicle assembly. This ECM formation and breakdown is essential for follicular development [37]. CTGF has previously been shown to affect ECM structure and stability in other tissues [36]. CTGF was primarily localized to the ECM of nest basement membranes and to the cell membranes of individual oocytes. These results are consistent with the general sub-cellular localization of CTGF in other tissues [36]. So one mechanism of action for CTGF may be for CTGF to affect ECM during follicle assembly, possibly promoting the cell migration that is a part of primordial follicle formation [37].

Ovaries were also treated for ten days in culture to investigate the possibility of manipulating the primordial follicle pool size at a time near the end of follicle assembly. In the event the number of assembled follicles could be affected with a growth factor treatment, observations would indicate the possibility of affecting the primordial follicle pool size and thus reproductive lifespan of a female. The nests found after 10 days of treatment were markedly smaller than nests after two days (Figure 2D). *In vivo* no unassembled nests would be observed after 10 days of postnatal development, but the ovary organ culture system has the capacity to retain small nests of unassembled oocytes. After 10 days of culture, CTGF was not found to increase the proportion of assembled follicles as was observed after two days of treatment. The likely explanation for this is that CTGF only increases the rate of follicle assembly. The control untreated ovaries appear to have “caught up” developmentally over the longer 10-day culture time period.

Interestingly, the combined treatment of both CTGF and TGFβ-1 was observed to increase the proportion of assembled follicles over ten days. This suggests that CTGF and TGFβ-1 act synergistically to promote follicle development since the CTGF treatment group alone did not have an effect after ten days of culture. This also indicates this treatment may affect follicle transition which takes place immediately after assembly. In contrast, progesterone was seen to inhibit follicle assembly and development over ten days [31]. Progesterone was found to increase the proportion of unassembled follicles, and the combined CTGF and TGFβ-1 treatment decreased the proportion of unassembled follicles. Therefore, the inhibitory effects of progesterone and stimulatory effects of the combined CTGF and TGFβ-1 treatment on primordial follicle assembly can be explained by their respective actions on the unassembled follicle pool, even without their having effects on the total number of oocytes. The separate actions of TGFβ-1 and CTGF on follicle assembly appear different than their combined effects. For example, TGFβ-1 alone decreased total oocyte number in 10-day ovary cultures, while TGFβ-1 plus CTGF did not. This suggests that their actions combined are not simply the additive result of their separate actions. It is likely that the synergism of signaling pathways from both CTGF and TGFβ regulate specific and novel cellular functions.

Observations suggest the expression of progesterone, CTGF and TGFβ expression are coordinated with each other. Previous studies of rat follicle assembly indicates progesterone treatment of ovaries resulted in a 2.7-fold change in CTGF mRNA expression, and a 1.6-fold change in TGFβ-3 expression [33]. In the current study microarray analysis revealed that CTGF treatment resulted in a small (1.1-fold) but statistically significant (p<0.05) increase in TGFβ-3 expression. In other organ systems it has been shown that CTGF and TGFβ-1 modulate each other's expression and actions [41], [42]. The signaling pathways that link the expression suggest the assembly-inhibiting actions of progesterone stimulates the expression of CTGF and TGFβ's that in turn can promote follicle assembly. Observations suggest the inhibition of follicle assembly may prime the factors that in turn stimulate assembly.

The total number of oocytes per ovarian cross-section was measured in the ten-day culture experiments. Interestingly, none of the treatments that affected the percentage of assembled follicles (i.e. CTGF and TGFβ-1, or P_4_) resulted in a significantly different number of oocytes when compared to controls. Therefore, the altered percentage of primordial follicles was due to corresponding alterations in the assembly of unassembled oocytes into primordial follicles and not from altered oocyte number. However, TGFβ-1 treatment was found to significantly reduce the total number of oocytes per cross section compared to controls. Observations indicate that TGFβ-1 reduced the total oocyte number, such that the actions of TGFβ may reduce the future capacity for follicular development. Although the postnatal alteration in oocyte number suggests the possibility of manipulating the primordial follicle pool and reproductive lifespan for a female, previous studies have suggested that at puberty the primordial follicle pool may normalize. For example, an increased number of assembled follicles caused by activin treatment prepubertally was reduced to normal levels during puberty [30]. Further studies are needed to determine if the mechanisms involved during puberty compensate for any earlier prepubertal primordial follicle pool size manipulation.

Investigation of the molecular events involved in CTGF induction of primordial follicle assembly used a microarray analysis of the ovarian transcriptome. The 76 genes regulated by CTGF impact numerous cellular processes with cell differentiation and cell cycle being prominent. The gene network identified suggested an extracellular growth factor (CXCL2), receptor signaling (GPR132 and CTNNB1), numerous signaling systems and transcriptional factors are all involved in primordial follicle assembly, Figure 7. Numerous potential signal transduction processes and families of transcription factors are regulated by CTGF, Supplemental Tables 1 and S1. Clearly the cytokine CXCL2 is a potential regulator of primordial follicle assembly that needs to be investigated. Further information is needed before specific pathways or transcription processes could be considered for therapeutic targets. However, the molecular actions of CTGF on primordial follicle assembly provide insights into this critical developmental process.

In summary, experiments using postnatal day 0 cultured rat ovaries were performed to determine the effects of CTGF and TGFβ-1 on primordial follicle assembly. CTGF alone was found to stimulate primordial follicle assembly. Treatment with CTGF and TGFβ-1 together was found to stimulate follicle assembly. Neither of these treatments were found to act through an apoptotic pathway or alteration in oocyte number. Interestingly, TGFβ-1 alone was found to decrease the number of oocytes and follicles over ten days suggesting the possibility that primordial follicle pool size may be manipulated. This treatment will need to be tested *in vivo* to determine if the reproductive lifespan of females can be manipulated. Understanding the processes of follicle assembly and follicle transition could lead to therapies to prevent premature ovarian failure or to the ability to manipulate the onset of menopause. The current study identifies CTGF as one of the few growth factors known to be involved in primordial follicle assembly, and TGFβ-1 as a factor that can reduce prepubertal follicle pool size.

## Supporting Information

Table S1 List of differentially expressed genes in rat P0-Ovary under in vitro CTGF treatment (76 genes).
(0.06 MB PDF)
