# Supplementary material for: Induction of Ovarian Primordial Follicle Assembly by Connective Tissue Growth Factor CTGF
Source: PLoS One. 2010 Sep 24;5(9):e12979. doi: 10.1371/journal.pone.0012979 (PMC2945314; doi:10.1371/journal.pone.0012979)
Supplement: Table S1 — List of differentially expressed genes in rat P0-Ovary under in vitro CTGF treatment (76 genes). (0.06 MB PDF) [file pone.0012979.s001.pdf]

Supplemental Table 1

List of differentially expressed genes in rat P0-Ovary under *in vitro* CTGF treatment (76 genes)

**Apoptosis**					
Gene Symbol	***GenBank_Reference Sequence***	***mean_ diff (CTGF-Con)***	***Ratio CTGF/ Con***	***Affymetrix Probe Set ID***	***Gene Title***
Rybp	NM_001107879	74	1.22	10864441	RING1 and YY1 binding protein
**Cell Cycle**					
Egr1	NM_012551	26	1.20	10800919	early growth response 1
**Cytoskeleton & ECM**					
Mybpc1	X90475	-14	0.82	10901574	myosin binding protein C, slow type
**Development**					
Id4	NM_175582	12	1.23	10797722	inhibitor of DNA binding 4
Tsx	NM_019203	8	1.32	10934509	testis specific X-linked gene
Vgll1	XM_001058701	6	1.31	10935600	vestigial like 1 (Drosophila)
**Epigenetics**					
Prmt2	NM_001025144	-31	0.84	10829591	protein arginine methyltransferase 2
**Growth Factors**					
Cxcl1	NM_030845	35	1.21	10775900	chemokine (C-X-C motif) ligand 1 (melanoma growth stimulating activity, alpha)
**Immune Response**					
LOC683127	XM_001064581	-32	0.77	10759581	similar to CD209a antigen
**Metabolism**					
Agxt2	NM_031835	14	1.54	10813646	alanine-glyoxylate aminotransferase 2
Ard1b	NM_001024742	12	1.20	10771545	ARD1 homolog B (S. cerevisiae)
Pdia5	NM_001014125	26	1.33	10751498	protein disulfide isomerase family A, member 5
**Proteolysis**					
Spink5l1	NM_001008874	4	1.22	10801471	Kazal type serine protease inhibitor 1
**Receptors & Binding Proteins**					
Ly49s6	NM_001009488	-7	0.79	10866146	Ly49 stimulatory receptor 6
Olr1226	NM_001000442	-10	0.84	10909154	olfactory receptor 1226
Olr46	NM_001001001	3	1.23	10709370	olfactory receptor 46
Olr630	NM_001001059	5	1.31	10847111	olfactory receptor 630
Olr684	NM_001001378	5	1.21	10837624	olfactory receptor 684
Olr690	NM_001000569	5	1.21	10847178	olfactory receptor 690
Olr727	NM_001000619	-5	0.76	10837638	olfactory receptor 727
Olr901	NM_001000057	-8	0.78	10893309	olfactory receptor 901
Olr920	NM_001001356	13	1.42	10900008	olfactory receptor 920
RGD1559730	AAHX01105426	-7	0.78	10757314	similar to putative pheromone receptor (Go-VN4)
Vom2r12	NM_001099488	-3	0.83	10703568	vomeronasal 2 receptor, 12
**Signaling**					
Cby1	NM_145676	-23	0.78	10897698	chibby homolog 1 (Drosophila)
Gpr132	NM_001170595	6	1.22	10892343	G protein-coupled receptor 132
MGC109340	BC092634	-25	0.83	10935036	similar to Microsomal signal peptidase 23 kDa subunit (SPase 22 kDa subunit) (SPC22/23)
Minpp1	NM_019263	20	1.28	10719148	multiple inositol polyphosphate histidine phosphatase 1
LOC690930	XM_001078213	-5	0.84	10728930	similar to membrane-spanning 4-domains, subfamily A, member 6B
**Transcription**					
Dnaja4	NM_001025411	7	1.24	10910084	DnaJ (Hsp40) homolog, subfamily A, member 4
Dnajc30	NM_001109024	-16	0.72	10761225	DnaJ (Hsp40) homolog, subfamily C, member 30
Hint1	BC168732	-7	0.77	10862836	histidine triad nucleotide binding protein 1
RGD1564534	BC111405	16	1.50	10933997	similar to CHCHD4 protein
**Translation & Protein Modification**					
Eif1	NM_001105837	13	1.20	10738170	eukaryotic translation initiation factor 1
Lin28	NM_001109269	31	1.21	10862855	lin-28 homolog (C. elegans)
LOC298509	XR_007384	-6	0.82	10879545	similar to 60S ribosomal protein L21
LOC688243	ENSRNOT00000045033	-6	0.82	10719182	similar to Nucleolin (Protein C23)
RGD1559877	XM_344657	10	1.20	10800511	similar to 60S ribosomal protein L29 (P23)
RGD1562381	AAHX01045217	-6	0.76	10890687	similar to ribosomal protein S17

**Miscellaneous & Unknown**					
Fam83b	NM_001108169	-5	0.83	10918770	family with sequence similarity 83, member B
LOC499144	ENSRNOT00000024645	-45	0.84	10706363	hypothetical protein LOC499144
LOC688531	AAHX01103576	-22	0.82	10779575	similar to Spetex-2C protein
LOC688531	AAHX01103576	-24	0.81	10779565	similar to Spetex-2C protein
**EST's**					
RGD1564937	NM_001134636	-5	0.81	10917293	similar to RIKEN cDNA 1110032A03
LOC366913	XR_007555	11	1.24	10903187	hypothetical gene supported by NM_012963
LOC500947	NM_001144870	-11	0.81	10908084	hypothetical gene supported by BC088439
	NC_001665	216	1.23	10930569	
	ENSRNOT00000056466	-101	0.74	10824788	
	ENSRNOT00000053884	-24	0.64	10804339	
	AAHX01034922	8	1.23	10867629	
	XR_005979	-4	0.82	10935242	
	rno-mir-15b	-24	0.78	10815913	
	GENSCAN00000046391	-5	0.80	10856213	
	GENSCAN00000023337	127	1.23	10820008	
	ENSRNOT00000061205	-19	0.70	10800513	
	ENSRNOT00000054186	-8	0.80	10887022	
	ENSRNOT00000054186	-8	0.80	10887038	
	ENSRNOT00000054183	-3	0.81	10787848	
	ENSRNOT00000053920	-17	0.79	10880159	
	ENSRNOT00000053920	-17	0.79	10880161	
	ENSRNOT00000053527	-5	0.80	10932910	
	ENSRNOT00000053115	-5	0.78	10722315	
	ENSRNOT00000052903	8	1.23	10903518	
	ENSRNOT00000052808	9	1.30	10933949	
	ENSRNOT00000051775	-8	0.72	10770827	
	ENSRNOT00000031920	6	1.29	10711399	
	AABR03116355	-5	0.84	10829618	
	ENSRNOT00000052215	9	1.30	10773706	
	ENSRNOT00000051003	-10	0.81	10734770	
	ENSRNOT00000000043	-4	0.83	10867491	
	---	43	1.59	10809904	
	---	-24	0.63	10799310	
	---	4	1.21	10818241	
	---	2	1.21	10729571	
	---	-7	0.75	10750926	
	---	-8	0.78	10937572	
